# Computed tomography osteoabsorptiometry for imaging of degenerative disc disease

**DOI:** 10.1016/j.xnsj.2022.100102

**Published:** 2022-02-12

**Authors:** Max Hans-Peter Gay, Gordian Born, Arne Mehrkens, Holger Wittig, Magdalena Müller-Gerbl

**Affiliations:** a Institute of Anatomy, Department of Biomedicine, Musculoskeletal Research, University of Basel, Pestalozzistrasse 20, 4056 Basel, Switzerland; b Department of Biomedicine, Tissue Engineering, University of Basel, Hebelstrasse 20, 4056 Basel, Switzerland; c Department of Spinal Surgery, University Hospital Basel, Petersgraben 4, 4031 Basel, Switzerland; d Institute of Forensic Medicine, University Basel, Health Department Basel-Stadt, Pestalozzistrasse 22, 4056 Basel, Switzerland

**Keywords:** Computed tomography -osteoabsorptiometry, Subchondral bone plate, Subchondral bone mineralization, Intervertebral disc, Degenerative disc disease, Low back pain, LBP, low back pain, SBP, subchondral bone plate, IVD, intervertebral disc, DDD, degenerative disc disease, CT-OAM, ct oseoabsorptiometry, HU, Houndsfield Units

## Abstract

**Background:**

Lower back pain is a common condition with significant morbidity and economic impact. The pathophysiology is poorly understood but is in part attributable to degenerative disc disease (DDD). The healthy intervertebral disc ensures spine functionality by transferring the perceived load to the caudally adjacent vertebrae. The exposure to recurring mechanical load is mirrored in the mineralization pattern of the subchondral bone plate (SBP), where increased bone density is a sign of repetitive localized high stress. Computed tomography -osteoabsorptiometry (CT-OAM) is a technique based on conventional CT scans that displays the mineral density distribution in the SBP as a surface-color map. The objective of this study was to measure and analyze the SBP mineral density patterns of healthy lumbar intervertebral disc (IVDs) and those suffering DDD using CT-OAM densitograms. These findings should provide in vitro insight into the long-term morphological properties of the IVD and how these differ in the state of disc degeneration.

**Methods:**

The CT-data sets of spines from 17 healthy individuals and 18 patients displaying DDD in the lumbar spine were acquired. Individual vertebrae of both cohorts were 3D reconstructed, processed using image analysis software, and compared to one another. Maximum intensity projection of the subchondral mineralization provided surface densitograms of the SBP. The relative calcium concentration, the local maxima of mineralization, and a mean surface projection of level-defined SBPs were calculated from the densitogram and statistically compared.

**Results:**

The inferior SBP, adjacent to degenerating disc, display an 18-41 % higher relative calcium concentration than their healthy counterparts. In the opposing superior SBPs the relative calcium content is significantly increased. Whereas it is reasonably consistent for L1-L3 (L1: 132 %, L2: 127 %, L3: 120 %), the increase grows in caudal direction (L4: 131 %, L5: 148 %, S1: 152 %). Furthermore, a change in the areal distribution of excessive mineralization can be differentiated between healthy and diseased motion segments.

**Conclusions:**

The acquired data provide in vitro proof of the mechanical and anatomical properties of the SBP in relation to the state of disc degeneration. In conjunction with the diagnostic use of CT-osteoabsorptiometry, our data provide a basis for a non-invasive and sensitive technique that correlates with disc functionality. This could be promising in various cases, from early identification of early stages of DDD, tracking disease progression, and assessing the repercussions of surgical procedures or experimental therapies.

## Introduction

At some point in their lives, 70 – 85 % of all people experience low back pain (LBP) [1]. The recovery rate lies around 90 % at three months [1]; however, an estimated 25 % develop recurrent LBP within a year after the resolution [2], and 10 – 15 % of all patients develop chronic pain and disability [3], [4], [5]. This burdens patients to the extent that it was the leading cause of years lived with disability in the 2013 Global Burden of Disease Study [6]. Thus, it puts a substantial strain on the healthcare systems.

Several conditions are known to provoke LBP. The leading and most strongly associated cause is degenerative disc disease (DDD) despite the limited knowledge of its pathophysiology [7]. In his literature review about LBP, Vlaeyen (2018) points out that: "It should also be kept in mind that disc, end-plate, vertebral and other structural changes observed from imaging might contribute to LBP, or alternatively might be markers of other conditions that lead to back pain" [8].

The healthy intervertebral disc (IVD) performs two contrary functions in combination with muscles and ligaments. On the one hand, it supports the stability of the spine; on the other hand, it is also responsible for the flexibility and agility of the spine. The IVD ensures these functionalities by transferring the cranial perceived load to the caudally adjacent vertebrae. Numerous studies have examined this transmission of load and have come to a collective conclusion; a healthy disc consistently distributes both perceived central or peripheral load onto the underlying vertebra thanks to its unique anatomy [9], [10], [11], [12], [13], [14], [15], [16], [17], [18], [19], [20].

The IVD consists of a gelatinous core, the nucleus pulposus, radially confined by the annulus fibrosus. The cartilage end-plates lie cranially and caudally and adjoin the neighboring vertebrae [21]. Axial load on a cranial end-plate leads to compression of the nucleus pulposus. The load is redirected radially to the annulus fibrosus exploiting it to shear stress and traction. The previously mentioned studies derived these conclusions utilizing computer simulation and invasive or detrimental procedures. These findings hold great importance, yet they only portrayed "snapshots" of independent mechanical loading scenarios. However, the various everyday movements, loads, and postures have individual effects, which accumulate over time and impact the entire spine.

A wide range of classification systems exists to grade DDD. The two predominant classification systems are the Pfirrmann scale, which considers the nucleus pulposus and the annulus fibrosus [22], and Modic changes, which consider degenerative end-plate changes [23,24]. Both systems are MR-based, offering a detailed and non-invasive evaluation of the state of the disc structure. However, the acquired radiological parameters give no readout to disc functionality.

The exposure to recurring mechanical load in a joint is mirrored in the various allocation of mineralization of the subchondral bone lamellae [25], [26], [27], [28], [29]. An area of increased bone density in a joint's subchondral bone plate (SBP) is a sign of repetitive localized high stress. Mineral density distribution in the SBP can be represented using CT-osteoabsorptiometry (CT-OAM), a reliable, non-invasive technique. CT-OAM is based on conventional CT scans using Hounsfield Units (HU). The HUs are converted to display mineral densitograms, which are surface-displayed in a false-color map on the joint surface. This method has provided the density patterns for several joints. In part, some studies included mechanical indentation techniques to correlate the densitogram values with the physical properties of the SBP [29], [30], [31], [32], [33], [34], [35], [36], [37], [38], [39].

The objective of this study was to measure and analyze the SBP mineral density patterns of healthy lumbar IVDs and those suffering DDD using CT-OAM densitograms. These findings should provide in vitro insight into the long-term morphological properties of the IVD and how these differ in the state of disc degeneration.

## Material and methods

### Cohorts

Thirty-four spines were evaluated, 17 from healthy individuals (15 male, two female, age range 19 - 40 with an average age of 27) and 18 from patients (6 male, 12 female, age range 27 - 67, with an average age of 47) displaying DDD in the lumbar spine on at least one level. The CT- data from the healthy cohort was contributed by the Institute of Forensic Medicine, University Basel, Health Department Basel-Stadt. The CT scans from the DDD group were provided from the data collection from the Institute of Anatomy at the Ludwig Maximilians-University Munich and the Department of Spinal Surgery of the University Hospital Basel.

A history of LBP was present in all DDD cases. Spines of the DDD were respectively diagnosed at the Department of Radiology at the Ludwig Maximilians-Universität Munich and the Department of Spinal Surgery of the University Hospital Basel. Cases with herniated discs were excluded as the functionality of the motion segment was considered to be compromised. Overall the subchondral mineralization pattern of 102 healthy (all six levels in each of the 17 healthy cases) and 49 degenerated discs were evaluated. The number of the degenerated cohort with respective levels was five at Th12 - L1, six at L1 - L2, six at L2 - L3, seven at L3 - L4, 15 at L4 - L5, and ten at L5 - S1.

The use of anonymous radiological images for research purposes is ethically and lawfully approved under the swiss Human Research Law article 2 paragraph 2a.

### Computed tomography osteoabsorptiometry

**Fig. 1:**
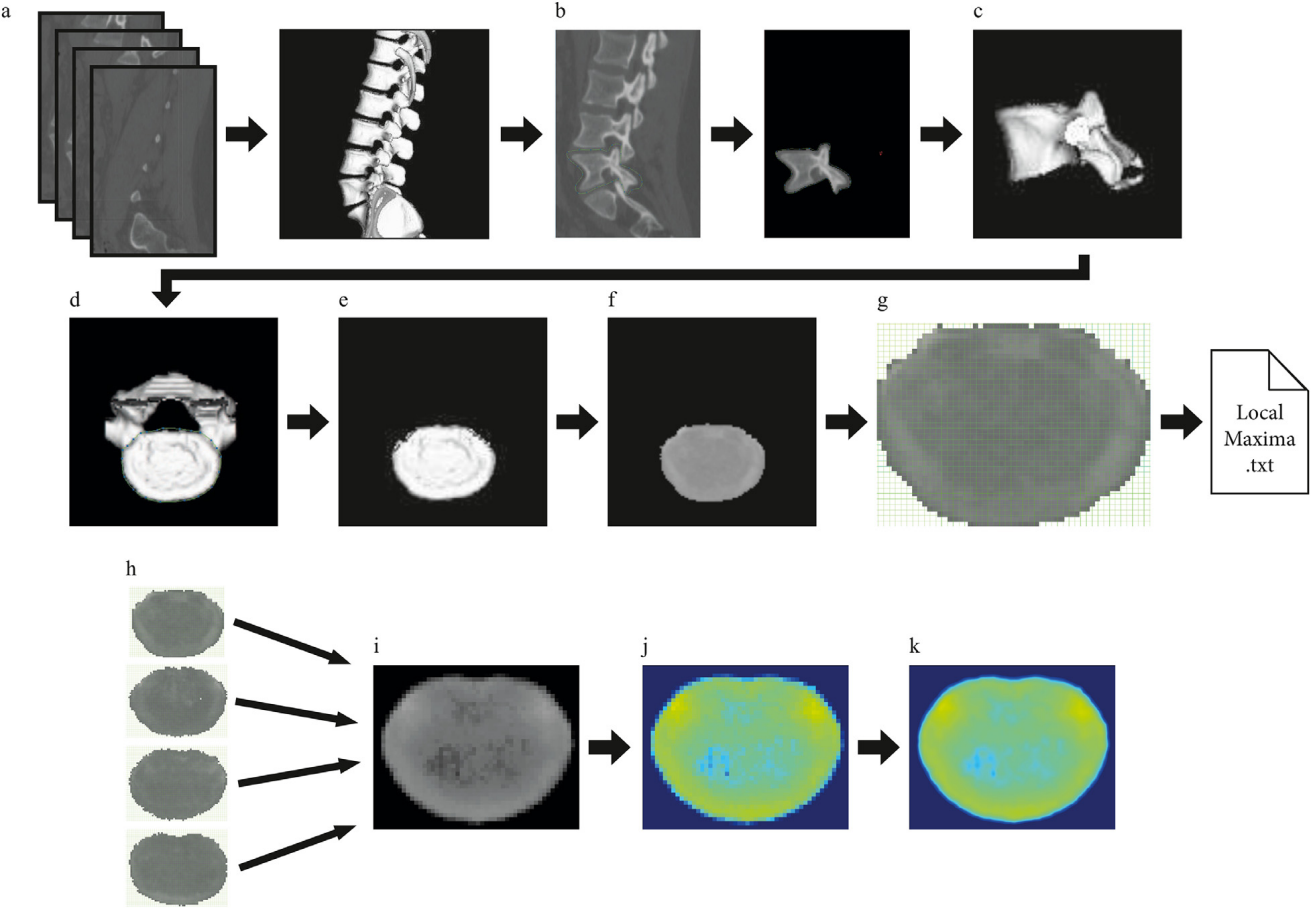
CT-OAM methodology a) CT-slices of 1 to 1.5 mm thickness were uploaded to the image analysis software ANALYZE ® 11.0. b) The relevant vertebra was manually outlined and cropped in individual CT scan slices. c) The slices were then reconstructed three-dimensionally and d) rotated to attain a perpendicular view of the desired joint surfaces. e) The subchondral bone plate was manually rendered. f) Greyscale values corresponding to the respective houndsfield unit were surface-projected using a Maximum Intensity Projection algorithm generating a greyscale densitogram. g) The densitograms were standardized by applying a coordinate raster of 50 × 40, which offered comparable calculation of the local maxima h) The rasterized densitograms of the subchondral bone plate of an individual vertebra i) were averaged to create a mean densitogram. j) A false color-code corresponding to the houndsfield units was applied and k) smoothened by bicubic interpolation.

Conventional clinical CT devices from Siemens (Munich, Germany) acquired data sets used for the evaluation of mineralization patterns employing CT-OAM (University of Munich: SOMATOM Plus 4 TM; University Hospital Basel: SOMATOM Definition AS and Flash; Institute of Forensic Medicine, University Basel: SOMATOM Emotion 16 Slice). CT-slices from Munich were coronal and 1 mm thick, whereas those from both facilities in Basel were 1,5 mm thick sagittal slices (Fig. 1a).

CT datasets were processed using the image analysis software ANALYZE ® 11.0 (Biomedical Imaging Resource, Mayo Foundation). Respective vertebrae were first separately outlined manually within the CT scans (Fig. 1b). After that, they were reconstructed three-dimensionally (Fig. 1c) and rotated to attain a perpendicular view of the individual joint surfaces (Fig. 1d). The SBPs of both end-plates were manually isolated and rendered (Fig. 1e). The Maximum Intensity Projection algorithm of the software determined the highest density values of the SBP, assigned a greyscale value corresponding to the respective HU, and projected it onto the surface resulting in a densitogram (Fig. 1f).

### Densitogram post-processing

To quantitatively compare individual SBP to one another relative calcium concentration, the local maxima of mineralization, and a mean surface projection were calculated from the densitogram.

Relative calcium values were calculated by dividing the absolute calcium content by the surface area of the SBP. Absolut calcium concentrations were derived from ascribing known calcium values to the corresponding HU. Calcium values were then integrated over the number of pixels designated to that specific calcium value. The surface area was calculated by integrating the number of pixels of the SBP over the resolution. The resulting quotient is then displayed as mg of calcium per milliliter of surface area (mg/ml).

Local maxima and mean surface projection were computed by an application programmed in python (Python Software Foundation). Pictures were separately loaded as multi-dimensional arrays (after this named "images") with respective greyscale vectors (pixels). The program laid a predefined grid of 50 × 40 over each densitogram of a specific SBP, and the "isMaxima" function determined their respective local maxima. The maxima values and their corresponding grid locations were saved in a text file (Fig. 1g). Additionally, the program standardized the size and resolution of each densitogram by creating a rasterized image according to the 50 × 40 grid. The new pixels of the rasterized image were the average of the pixel parts inside the grid segments of the original image. The rasterized images of a specific SBP (Fig. 1h) were then overlaid and averaged to create a mean densitogram of the desired SBP (Fig. 1i). To better display, the mineral density distribution of the densitogram, these data were false color-coded using the jet color map to correspond with the respective HU (Johnston et al., 2010) with values chosen to be ≤ 800 to ≥ 2200HU (Fig. 1j). The mean densitogram was then bicubically interpolated to smoothen the image. (Fig. 1k)

### Analyses of densitogram patterns and local maxima

The median and standard deviation of relative SBP calcium values were plotted. Statistical comparison was assessed by a two-way ANOVA test with Sidak's multiple comparison post-test. Statistically significant was considered with p < 0.05 (Prism 9, Graph Pad Software).

The coordinates and HU of the acquired local maxima were transferred to a bubble plot corresponding to the grid using Prism 9 (GraphPad Software). If more than 20 local maxima were determined for a single image, only the highest 20 were plotted to the graph. The mineral density patterns were evaluated based on the appearance of the mean densitogram for the individual SBP of a specific level. High mineralization zones were defined as > 1000 HU. Accentuation of the high mineralization zones was visualized through a black-white image with a threshold cut off at 1000HU performed in Adobe Photoshop CC 2018. The regions with high HU values were described based on anatomical location.

## Results

### Higher calcium concentration in SBP of degenerated IVDs

**Fig. 2:**
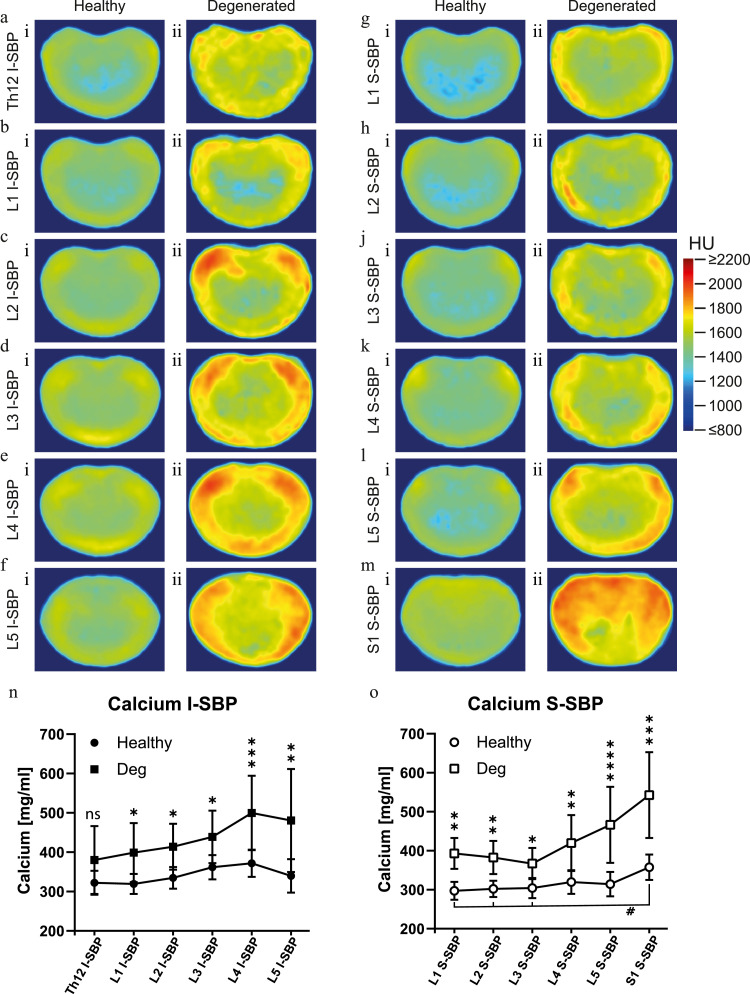
Subchondral calcium concentrations of healthy spines versus those with DDD CT-OAM averaged densitograms of a-f) the inferior subchondral bone plate (I-SBP) of vertebrae Th12 through L5 and g-m) the superior subchondral bone (S-SBP) of L1 through S1 of i) healthy spines and ii) those suffering from degenerative disc disease. Calcium concentration of healthy (circle) and degenerated (square) n) I-SBP and o) S-SBP in mg of calcium per milliliter of surface area (mg/ml).

In a first evaluation of the densitogram, more pronounced HU scores were apparent in all EPs of degenerated IVDs compared to EPs of healthy discs independent of the disc level (Fig. 2 a - m). This indicates higher levels of mineralization in the SBP of degenerated discs. The calcium concentration was integrated over the volume to allow a quantitative comparison. The superior EPs of the lumbar vertebrae with healthy discs showed a calcium concentration between 300 and 320 mg/ml throughout all levels. However, most caudally, a maximal SBP calcium concentration is seen for S1 (358 ± 33 mg/ml), which was significantly higher than values for L1 through L3 (Fig. 2o). The calcium value of the inferior SBP was virtually the same for Th12 (322 ± 31 mg/ml) and L1 (319 ± 26 mg/ml). In the following vertebrae, the value increased in caudal direction with values of 335 ± 28 mg/ml for L2, 362 ± 31 mg/ml for L3, and 372 ± 35 mg/ml for L4, after which reduced calcium concentrations were seen in the inferior SBP of L5 (340 ± 42 mg/ml).

As stated previously, the relative calcium concentration of SBP of degenerated IVDs differs. The gradual increase of calcium content in the inferior SBPs from Th12 in caudal direction until L4 with a decrease at L5 was also distinguishable in diseased spines except for being at a significantly higher level (Th12: 380 ± 86 mg/ml, L1: 399 ± 75 mg/ml, L2: 414 ± 58 mg/ml, L3: 439 ± 67 mg/ml, L4: 500 ± 95 mg/ml, and L5: 481 ± 131 mg/ml; Fig. 2n). Except for Th12, these values are significantly higher than in healthy cases with increased values of 118 % for Th12, 124 % for L1, 124 % for L2, 121 % for L3, 134 % for L4, and 141 % for L5.

Although the values for the superior SBP adjacent to degenerated disc are also significantly higher than those of healthy discs, they display an altered cranial to caudal calcium content profile. In healthy conditions, a relative consistent calcium content was measured for all the superior SBP of the vertebra up to and including L5. However, in a degenerated spine, a minimal nonsignificant decrease is observed from L1 (393 ± 40 mg/ml) to L3 (367 ± 40 mg/ml), which changes to a distinct increase in L4 (420 ± 72 mg/ml) and a significant increase in L5 (466 ± 98 mg/ml), and S1 (542 ± 110 mg/ml) compared to their cranial lying vertebrae (Fig. 2o). This is also mirrored in the relative increase from healthy to degenerated conditions L1: 132 %, L2: 127 %, L3: 120 %, L4:131 %, L5:148 %, and S1: 152 % (Fig. 2o).

**Fig. 3:**
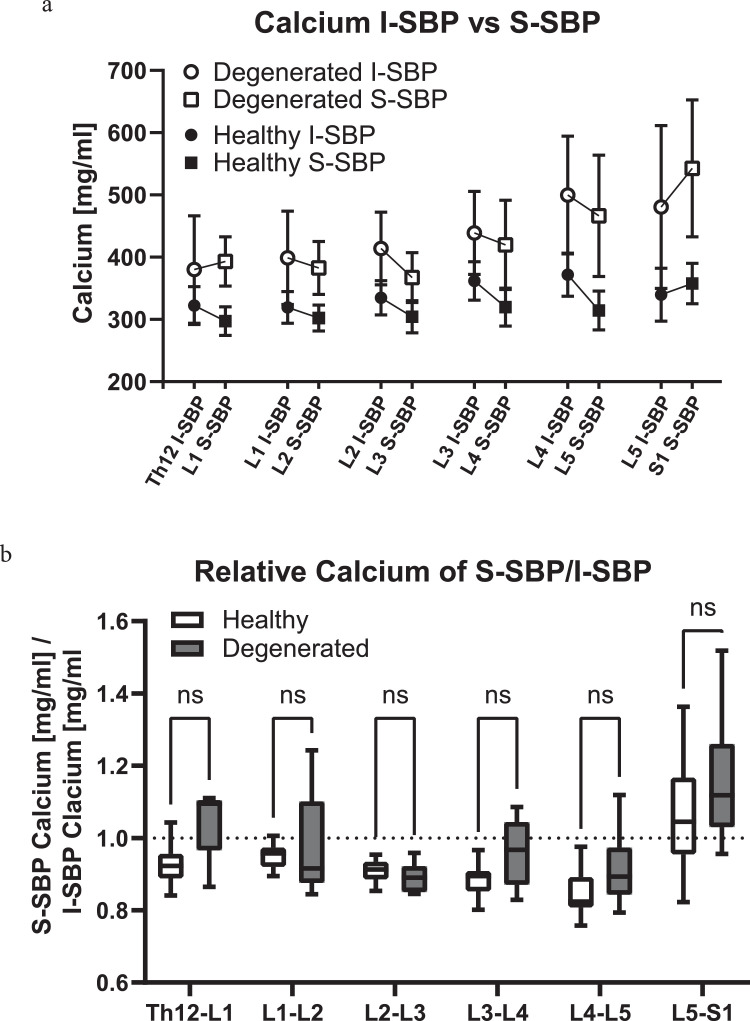
Relative calcium concentration comparison between healthy and diseased motion segments a) Calcium concentration in mg of calcium per milliliter of surface area (mg/ml) of inferior subchondral bone (I-SBP) and superior subchondral bone (S-SBP) of separate healthy and degenerated motion segments of the lumbar spine. b) Relative calcium concentrations of S-SBP vs. I-SBP of the respective healthy and diseased intervertebral joints of the lumbar spine

Consistent behavior between the inferior SBP versus superior SBP calcium content ratio could be demonstrated for vertebrae Th12 through L5 in healthy spines. The calcium content of SBP cranially to the IVD is always higher than the caudal lying SBP for Th12/L1 to L4/5 (Fig. 3a). This relationship is not only represented in the average concentrations but is also seen in the ratio between the inferior and superior SBP of the same individual (Th12/L1: 0.93 ± 0.05, L1/L2: 0.95 ± 0.03, L2/L3: 0.91 ± 0.03, L3/L4: 0.89 ± 0.04, L4/L5: 0.85 ± 0.06; Fig. 3b).

Interestingly, these observations differ in the SBP of the movement segment of L5/S1. The average superior SBP of S1 has a slightly higher concentration than the average inferior SBP of L5. This inverse in the relationship comes from a higher value for the superior SBP of S1 than the virtually constant value present in L1-L5, and a surprising decrease in the calcium value for I-SBP L5 in the otherwise increasing trend seen from Th12 to L4 (Fig. 3a). This higher ratio was also observed in a majority of the cases, where the relationship of SBPs from the same individual was compared (L5/S1: 1.07 ± 0.15, Fig. 3b).

Interestingly, the ratios of superior to inferior SBPs found in degenerated movement segments L1-L2 through L5-S1 were complementary to those seen in healthy individuals (L1/L2: 0.98 ± 0.15, L2/L3: 0.89 ± 0.04, L3/L4: 0.96 ± 0.09, L4/L5: 0.91 ± 0.10, and L5/S1: 1.07 ± 0.15). Surprisingly, the SBPs Th12 and L1 flanking a degenerated disc display an inversed relationship (Th12/L1: 1.05 ± 0.10) compared to those of healthy IVDs, however not to a significant degree (Fig. 3b).

### Averaged areal distribution of excessive mineralization

**Fig. 4:**
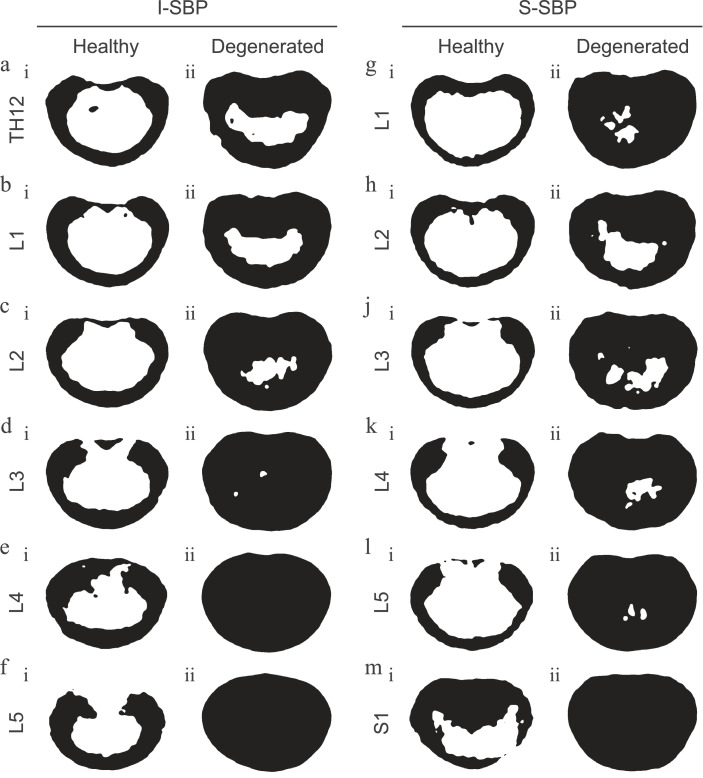
Alterations in mineralization patterns Averaged areal distribution patterns of mineralization for values ≥ 1000 HU of a-f) the inferior subchondral bone (I-SBP) of vertebrae Th12 through L5 and g-m) the superior subchondral bone (S-SBP) of L1 through S1 of i) healthy spines and ii) those suffering from degenerative disc disease.

Typical areal distribution patterns of mineralization were determined by averaging the level-specific densitograms and performing a threshold cut-off for values under 1000 HU. An indistinguishable pattern can be observed in both the superior and inferior SBP of the top five motion segments of the lumbar spine. This structural pattern can be described as a continuous peripheral ring with pronounced domains in the dorsal lateral region anterior to where the pedicles emerge. Moving medially from these domains, the ring breadth diminishes and, in some cases, even dissipates only to reemerge and thicken again in the dorsal median (Fig. 4a.i - e.i and g.i - l.i).

Interestingly, the mineralization area profiles between L5 and S1 deviate from this pattern. The pattern of the inferior SBP of L5 is similar to those of the other lumbar vertebrae; however, it is void of the peripheral dorsal median field (Fig. 4 f.i). Surprisingly, the SBP of S1, opposite L5, has a pronounced dorsal median region that reaches halfway over the disc to the ventral side (Figure m.i).

When comparing the healthy areal mineralization patterns with those of SBP adjoining degenerative discs, two phenotypes could be distinguished between the top three-movement segment patterns versus the bottom three. Both the inferior and superior SBP of the top three joints showed a general thickening of the peripheral rim. This was most dominant in the dorsal median region, where the area was minimally apparent in healthy conditions; however, it almost extended until the center of the plate in the occurrence of disc degeneration (Fig. 4a.ii - c.ii and g.ii - j.ii). On the other hand, areal patterns of high calcification values are virtually indistinguishable from the actual appearance of whole SBP on either side of diseased discs in the lowest three levels (Fig. 4d.ii - f.ii and l.ii - m.ii).

### Local maxima of calcium accumulation

Information of the singular individuals might be reduced in the averaging process to the extent that it is unrecognizable. Given this, the depiction of local maxima values provided further insight complementing the areal distribution of mineralization. The local maxima of mineralization of the separate images were computed, plotted respective of their location, and color-coded according to their value.

In healthy spines, the patterns arising from the overlayed SBP local maxima of individual joints are similar to the patterns of the averaged areal distribution. However, the peripheral ring, which varies in thickness depending on the anatomical location in the averaged areal distribution, is a fairly uniform structure in the plot of the local maxima. Additionally, single local maxima with comparable values to points on the rim were found in the center of the SBP, predominantly in the dorsal sector.

**Fig. 5:**
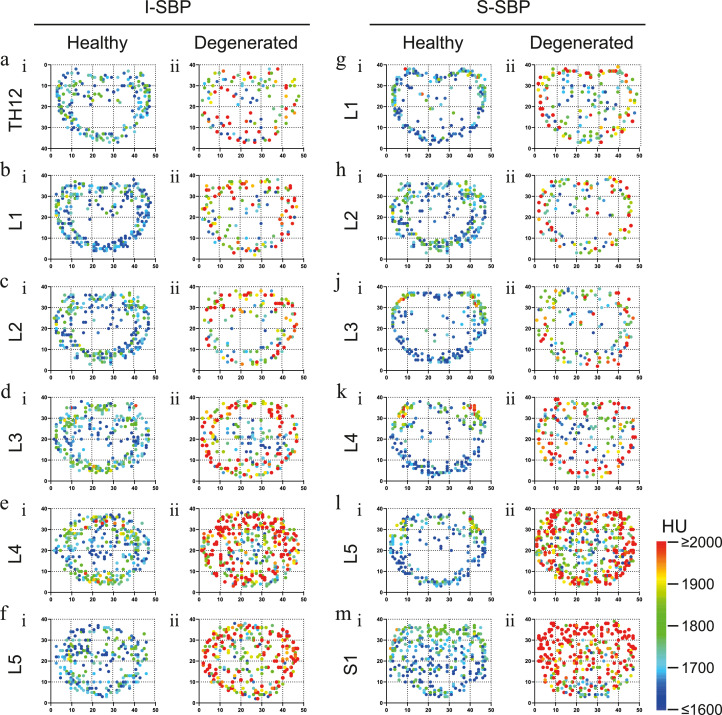
Areal distribution of local maxima of mineralisation Coordinates of the houndsfield unit color-coded local maxima of mineralization of a-f) the inferior subchondral bone (I-SBP) of vertebrae Th12 through L5 and g-m) the superior subchondral bone (S-SBP) of L1 through S1 of i) healthy spines and ii) those suffering from degenerative disc disease.

An exception to these findings is the joint surface of S1 in which the maxima are distributed over the entire SBP (Fig. 5a.i - m.i). Furthermore, the false color-coding showed that in the inferior SBP of L3 and L4, the highest maxima were found in the dorsal lateral regions as well as around the dorsal and ventral median (Fig. 5d.i-e.i), which coincides with the averaged densitograms (Fig. 2d.i - e.i). The superior SBPs of L3 - L5, on the other hand, only display the highest maxima values dorsal laterally; however, they lack distinctive high maxima in both peripheral medians (Fig. 5j.i - l.i), which also corresponds to the respective averaged densitograms (Fig. 2 j.i - l.i).

Local maxima patterning of mineralization in SBP neighboring degenerated disc retain a similar localization pattern as SBP of healthy discs in the top three joints; however, the highest maxima are found in the lateral margins (Fig. 5a.ii - c.ii and g.ii - j.ii). In the SBP of degenerative IVD L3/L4 to L5/S1, more local maxima were present in the central region (Fig. 5d.ii. - f.ii and k.ii - m.ii). The highest maxima were again located along the lateral border for inferior SBP L3 through L5 and superior SBP L3 (Fig. 5d.ii - f.ii and j.ii). Moreover, the superior SBP of L5 and S1 have their highest value in the dorsal lateral region; however, L5 also has substantial high values ventrally (Fig. 5l.ii – m .ii).

## Discussion

This study quantified the subchondral bone density of intervertebral joint surfaces in healthy spines and those suffering from DDD. The resulting data were compared and provided insight into the perceived changes in load on the vertebrae brought on by disc degeneration.

### Sample selection

The spine can already display the first signs of degeneration in the second and third decades of a lifespan [40], [41], [42]. The pathological indication can be very subtle to virtually non-existent in an otherwise healthy individual but can nonetheless lead to pain. Further studies have shown that bone density and trabecular structure considerably decrease after the age of 40 [43,44]. For this reason, we aimed to evaluate preferably young individuals.

The age of patients suffering from DDD ranged from 27 - 67, with an average age of 47. The age range for this cohort was so wide due to two reasons. First of which was the aim to evaluate all intravertebral motion segments of the lumbar spine. Secondly, it is rare for physicians to prescribe a computer tomography to diagnose DDD. These factors strongly reduced the number of possible patients from which to choose.

Acquisition of computer tomography scans from living healthy individuals was practically impossible due to the unwarranted radiation exposure. To our benefit, we could strictly choose our cohort by age and the lack of degenerative traits from the assortment of scans that the Institute of Forensic Medicine of the University Basel had to offer.

The predominant sex of the healthy (15 male, two female) and degenerative (6 male, 12 female) cohort were dissimilar and could raise the concern how the EPs of men and women compare. Although it has been established that male end-plates are 10 % larger than females, it has also been shown that the form and structural composition are identical in both genders [45]. Furthermore, when we compared our data of healthy males with that of healthy women, no imminent differences could be determined (data not shown). In this regard, we believe the cohorts are comparable for our data's readout to represent effects brought forward by disc degeneration.

### Load transfer in the spinal segments between Th12 and L5

Our analysis of healthy spines showed that the relative calcium content of the inferior SBP gradually increases from L1 to L4. This is not surprising as the absolute load on the vertebra increase in the caudal direction due to the growing weight of the body. Interestingly, we saw that the superior SBP is consistent for the lumbar vertebrae. In addition, reduced calcium content was observed when the cranial SBP were compared to the caudal SBP of the same motion segments (Th12/L1: 7 %, L1/L2: 5 %, L2/L3: 9 %, L3/L4:11 %, and L4/L5: 15 %). The findings predicate that the lumbar IVDs between Th12 and L4 absorb an extent of energy.

For a long time, it was postulated that the IVD distributes perceived loads onto the entire surface of the underlying end-plate in a uniform manner. This concept was put into question by a study in which pressure sensors were placed into healthy IVDs. This revealed a constant homogenous pressure in the central nucleus pulposus; however, significantly higher values were measured in the annulus fibrosus. [11].

If discs were to transfer the load onto the caudal end-plate uniformly, we would expect to see a homogenous joint surface when considering that bone morphology and mineralization adapt to load. However, our evaluation of respective densitograms and the localization of density maxima displayed an excess of calcium content in the periphery of the plate with larger areas and higher values in the dorsal lateral region. These results support the notion of a higher load beneath the annular fibrosus along the rim of the disc. This area corresponds to the annular epiphysis, which is where the collagen fibers of the annulus fibrosus integrate into the marginal ridge of the vertebra.

This assumption is further supported by studies that looked at stress distribution on the vertebrae and IVDs under different physiological movements using the finite element method (FEM) [46], [47], [48]. This work showed that the maxima of stress and strain were localized at the dorsal lateral region and in the ventral median region of both IVD and vertebra. Furthermore, the stress and strain on the vertebrae were higher in the inferior surfaces than on the superior surfaces of the caudal lying vertebra.

Taken together, our data, pressure sensor data, and FEM-models suggest that the initial pressure applied to a disc is reduced when transferred to its caudal SBP as the stretching of the annulus fibrosus partially absorbs energy.

### Motion segment L5/S1

In contrast to the lumbar spine's other intervertebral joints, the L5/S1 motion segment displays equal if not higher relative calcium content in its cranial SBP than in the caudal SBP. Both of the values opposed the developing trend that was observed in the cranial to caudal direction. Furthermore, as the pattern of excess mineralization for the inferior SBP of L5 resembles its counterparts on the cranial lying vertebrae, the SBP of S1 has an entirely distinct pattern.

The pattern of the inferior SBP of L5 is also pronounced in the periphery; however, it is void of excessive mineralization in the dorsal median area, which gives it the appearance of a horseshoe. On the other hand, the superior SBP of S1 shows a disproportionate mineralization region from the dorsal median rim to the center of the disc. A previous study showed that the SBP of the sacrum has a higher solidity, suggesting higher mineralization density, than the SBPs of vertebrae [49], which is in line with our findings.

For one thing, the sacrum is the base of the mobile spine, which lacks dampening elements caudally. A further difference between the lumbosacral SBPs and those found between the lumbar vertebrae is likely the consequence of the lumbar spine physiology at the lumbosacral transition, which displays a lordosis in the natural standing position. For one thing, this puts it under high amounts of bending stress.

The IVD of L5/S1 is shaped like a wedge. Together with the thick ventral fibers of the anterior longitudinal ligament, it functions as a deviation system, which transforms bending stress into compressive and tensile forces [50]. In addition to this, the resulting gravitational force of the natural standing position situates over the dorsal area of the joint surface, which leads to frequent propagation of load in this region. Although the lordosis is present in the lumbar vertebrae, it is strongly pronounced in L5/S1, and we believe this is the reason for the variance compared to the other motion segments.

### Effects of degenerative disc disease

As DDD progress, the water content of the nucleus pulposus diminishes, and it loses its capacity to translate the axial compression into shear and tensile forces on the annulus fibrosus. For the motion segment, the reasonable consequence of this would be that axial load on a disc is transferred to the superior SBP of the underlying vertebra without dampening. With respect to Wolff's law, the higher load would lead to higher mineralization, such as reflected in our data.

The inferior SBP, adjacent to degenerating disc, display an 18-41% higher relative calcium concentration than their healthy counterparts. The opposing superior SBPs also have more significant relative calcium concentration levels. The relative calcium content is reasonably consistent for L1 - L3 (L1: 132 %, L2: 127 %, L3: 120 %); however, the increase grows in caudal direction (L4: 131 %, L5: 148 %, S1: 152 %).

In the further aftermath of degeneration, the extra-axial forces can only partially be converted into shear and tensile force, which leads to an increased force transmission over the annular epiphysis [16,18,[51], [52], [53]]. This can also be seen in our results, where the outer ring of excessive mineralization is decidedly broader in vertebrae of degenerated spines, partially to the extent of covering the whole SBP.

## Conclusion

CT-OAM makes it possible to three-dimensionally visualize the distribution of the SBP density in a living individual in a non-invasive manner. We could show that values are consistent for healthy individuals and that apparent differences are distinguishable between SBP of healthy IVDs and those suffering from DDD.

These observed changes due to degeneration coincided with the results of studies that used FEM calculations or invasive and detrimental procedures. In this regard, CT-OAM offers a good insight into the joint of living individuals. The application of a non-invasive and sensitive technique that correlates with disc functionality could be promising in various cases. Including but not restricted to: ➔

• identifying early stages of DDD that would warrant pre-emptive conservative therapies
• tracking time-dependent changes in respect to disease progression in patients
• assessing the repercussions of surgical procedures (spinal fusion or implant of disc prosthesis) on neighboring joint segments

Furthermore, it is reasonable to expect that if the method can differentiate disease progression that it would also show recovery. Regenerative cell therapies are still experimental treatments, which have had inconclusive results in regard to quantitive MR imaging. CT-OAM could be a useful tool in evaluating the outcome of experimental regenerative therapies by assessing the recovery of disc functionality, which will be the focus of future work.

## Author contribution

Max Gay: Conceptualisation, Methodology, Data curation, Formal analysis, Writing - original draft. Gordian Born: Software programming, Arne Mehrkens: Data selection from Spinal Surgery and radiological evaluation Holger Wittig: Data acquisition and selection from forensic medicine, Magdalena Müller-Gerbl: Conceptualisation, Methodology, Software, Data curation from Ludwig Maximilians- University Munich, Writing - review & editing.

## Funding disclosure statement

There was no financial funding provided, outside of the research funding supplied by the university assosciated to the tenure of Prof. Müller-Gerbl. None of the authors have a study-specific appraisal of potential conflict of interest-associated bias.

## Declarations of Competing Interests

The authors declare that they have no known competing financial interests or personal relationships that could have appeared to influence the work reported in this paper.
